# Potential inhibitors designed against NDM-1 type metallo-β-lactamases: an attempt to enhance efficacies of antibiotics against multi-drug-resistant bacteria

**DOI:** 10.1038/s41598-017-09588-1

**Published:** 2017-08-23

**Authors:** Asad U. Khan, Abid Ali, Danishuddin, Gaurava Srivastava, Ashok Sharma

**Affiliations:** 1 Interdisciplinary Biotechnology Unit, Aligarh Muslim University Aligarh, Aligarh, 202002 India; 2 Biotechnology Division, CSIR-CIMAP, Lucknow, 226015 India

## Abstract

NDM-1 and its variants are the most prevalent types of metallo-β-lactamases, hydrolyze almost all antibiotics of β-lactam group leading to multiple-drug resistance in bacteria. No inhibitor has yet been obtained for NDM-1 or other class of metallo-β-lactamases. Therefore, strategies to identify novel anti-β-lactamase agents with specific mechanisms of action are the need of an hour. In this study, we have reported the discovery of novel non-β-lactam inhibitors against NDM-1 by multi-step virtual screening approach. The potential for virtually screened drugs was estimated through *in vitro* cell assays. Five chemical compounds were finally purchased and evaluated experimentally for their efficacies to inhibit NDM-1 producing bacterial cells, *in vitro*. The dissociation constants (Kd), association constant (Ka), stoichiometry (n) and binding energies (ΔG) of compounds with the respective targets were determined using isothermal titration calorimetry (ITC). Molecular dynamic simulation carried out for 25 ns revealed that these complexes were stable throughout the simulation with relative RMSD in acceptable range. Moreover, Microbiological and kinetic studies further confirmed high efficacies of these inhibitors by reducing the minimum inhibitory concentration (MIC) and catalysis of antibiotics by β-lactamases in the presence of inhibitors. Therefore, we conclude that these potential inhibitors may be used as lead molecules for future drug candidates.

## Introduction

Multi-drug resistance has become a major concern worldwide after the emergence of New Delhi metallo-β-lactamase-1 (NDM-1). The β-lactam antibiotics have long been a cornerstone for the treatment of bacterial disease. But β-lactamases are the most widespread cause of bacterial resistance to β-lactam antibiotics^1^. Bacteria use β-lactamase enzymes as a defence mechanism which hydrolyzes the amide bond in the β-lactam ring, yielding an inactivated product. The breakdown of β-lactam antibiotics by β-lactamases is the important mechanism of Gram negative bacteria against these drugs. Based on the Ambler classification scheme, β-lactamases are divided into four distinct classes (class A-D): three classes (A, C and D) are of serine hydrolyses whereas class B are metallo-β-lactamases (MBLs) which use one or two zinc ions in the active site to mediate hydrolysis^2^. Class-B enzymes are further divided into subclasses B1, B2 and B3. Of which the class B1 enzymes have emerged as the most clinically significant and are characterized as to have two active site for zinc ions. Metallo-β-lactamases (MBLs) have been found in all enterobacteriacae members^3, 4^. VIM and IMP enzymes are the most frequently circulating types of class B β-lactamases^5^.

NDM-1 has been identified as a new metalo-β-lactamase isolated from a Swedish patient who acquired urinary tract infection caused by *Klebsiella pneumoniae* ^6^. After the discovery of NDM-1, its variants are emerging worldwide, prompting the World Health Organization to issue a global warning^4^. This enzyme confers a broad spectrum β-lactamases resistance, hydrolyzing penicillins, cephalosporins and carbapenems. Moreover, most plasmids that harbour the NDM-1 gene often associated with other resistance markers, such as, quinolones, aminoglycosides, rifampin and sulfonamides, which makes NDM-1 positive strain resistant to multiple drugs. Therefore, the spread of NDM-1 producing bacterial strains is a serious worldwide menace.

One of the strategies to increase the efficacies of existing antibiotics against beta-lactamases is designing inhibitors^7^. Although several inhibitors have been reported earlier against NDM-1 but our hypothesis is different to design non-beta-lactam inhibitors so that chances of resistance is minimised^8^.

In view of the above background we intended to screen potential inhibitors against these metallo-**β**-lactamases and to further validate their efficacies against multi-drug resistant bacterial strains.

## Results

### Virtual screening against NDM-1

Five compounds were screened from Maybridge database with higher GOLD fitness score and binding energies as compared to the known inhibitors, selected for further study (Figures S1 and S2). The highest GOLD fitness scores of 117.55 and 116.54 were found for compounds, AW01220 and BTB02323, respectively (Table 1). Whereas, compounds, AW01120, RF01991 and HAN00094 made stable complex with GOLD fitness score of 109.17, 111.10 and 112.43, respectively. The binding energies predictions from AutoDock Vina were also high for these compounds. Compounds having reasonable diversity from the reference molecules were selected for experimental evaluation. Figure S2 shows the hierarchical clustering of compounds which are ranked based on docking score. Tanimoto coefficient score of these molecules lies in the range of 0.19–0.31 in comparison with reference molecule (Table 1).

**Table 1:** GOLD fitness **s**cores and binding energies of selected five inhibitors for NDM-1.

Molecule	Structure	Score	Xscore (kcal/mol)	Autodock (kcal/mol)	T.C
Mercaptocarboxylate		90.13	−7.68		1
BTB02323 (M17)		117.55	−8.42	−7.6	0.19
AW01220 (M1)		116.54	−8.57	−8.1	0.18
HAN00044 (M75		112.43	−8.53	−8.7	0.31
RJF01991 (M 21)		111.10	−8.83	−7.9	0.22
AW01120^61^		109.17	−8.45	−8.6	0.29

### Screened compounds

#### BTB02323 (M17)

**Figure 1:**
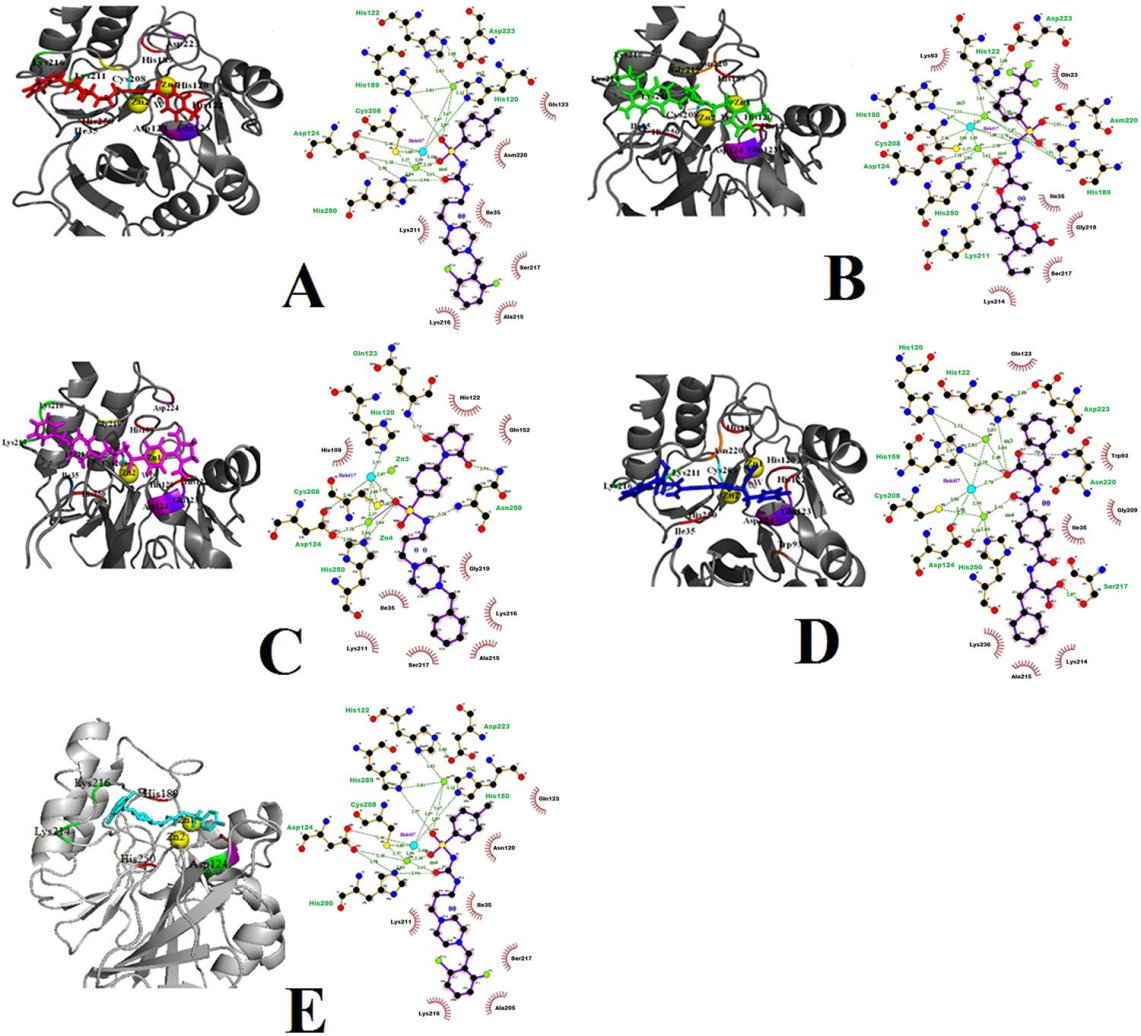
The predicted binding modes between the target enzyme and the compounds. (**A**) BTB02323(M17). (**B**) RJF01991(M21). (**C**) AW01120(M61). (**D**) HAN00044. (**E**) AW01120(M1).

This compound bind within the active site of NDM-1 with a GOLD fitness score of 117.55 and has a binding affinity of −8.42 kcal/mol from X-Score (Table 1). It formed two hydrogen bonds with the amino acids, His189 and His250 with the distance of 2.97 and 2.94, respectively (Fig. 1A). Oxygen atoms (O8 and O13) of this compound showed hydrogen bond interactions with these amino acids. Total 44 hydrophobic contacts were exhibited by Ile35, His122, Gln123, Asp124, His189, Lys211, Ala215, Lys216, Ser217 and His 250 within the range of 3.42–3.77 (Table 2). Highest hydrophobic interactions were shown by residue, Lys216 which was found to be involved in 10 interactions. While, amino acids; His122, Gln123 and His250 were found to form five hydrophobic contacts.

**Table 2:** NDM-1 hydrogen bonding and hydrophobic interactions.

Compound	Hydrogen Bonding		Hydrophobic Interactions	
	AminoAcids	Distance (Å)	Amino Acids	Distance (Å)
BTB02323	His189, His250	2.94–2.97	Ile35, His122, Gln123, Asp124, His189, Lys211, Ala215, Lys216, Ser217, His250	3.42–3.77
RF01991	His122, His189, Lys211, Asn220	2.50–3.10	Ile35, Trp93, His122, Gln123, Asp124, His189, Cys208, Lys211, Ala215, Lys216, Ser217, Gly219, Asn220, His250	3.05–3.90
AW01220	Gln123, Cys208, Asn220, His250	2.74–3.28	Ile35, His122, Gln123, Asp124, Glu152, His189, Lys211, Ala215, Lys216, Ser217, His250	3.20–3.85
HAN00094	His122, His189, Ser217, Asn220	2.84–3.29	Ile35,Trp93,His122, Gln123, Asp124, His189, Lys214, Ala215, Lys216, Ser217, Gly219, His250	2.88–3.90
AW01120	Lys211, Asn220	3.00–3.16	Ile35, Met67, Trp93, His122, Gln123, Asp124, His189, Cys208, Lys211, Ala215, Lys216, Ser217, Gly219, Asn220, His250	3.00–3.87

#### RJF01991(M21)

The binding energy from X-Score for this compound was calculated as −8.83 (Table 1) and with GOLD fitness score, was found 111.10. Four hydrogen bonds were observed among His122, His189, Lys211 and Asn220 residues within the range of 2.50–3.10 (Fig. 1B). In addition, fifty five hydrophobic interactions were involved in stabilizing the complex. Amino acids; Ile35, Trp93, His122, Gln123, Asp124, His189, Cys208, Lys211, Ala215, Lys216, Ser217,Gly219, Asn220 and His250 were involved in these hydrophobic interactions within the range of 3.05–3.90 (Table 2). Highest hydrophobic contacts were exhibited by Asp124 and Lys216, each of them was interacted with seven contact groups.

#### AW01220 (M1)

This compound made a complex with GOLD fitness score 116.54 and has a binding affinity −8.57 kcal/mol calculated from X-Score (Table 1). This complex was found to have five hydrogen bond interactions with Gln123, Cys208, Asn220 and His250 by the distance of 2.74 and 3.28 (Fig. 1C). Asn220 formed two hydrogen bonds. A total of seventy hydrophobic contacts were shown by Ile35, His122, Gln123, Asp124, Glu152, His189, Lys211, Ala215, Lys216, Ser217, His250 residues within the range of 3.20–3.85 (Table 2). Highest hydrophobic interactions were exhibited by His122 and Lys216, each of them was involved in eighteen and thirteen contacts, respectively. Whereas, amino acids Ser217, Asn220 and His250 were found to make five hydrophobic contacts.

#### HAN00044 (M75)

This compound made a complex with a GOLD fitness score of 112.43 through lower binding affinity, −8.53 kcal/mol (Table 1). It was found to have four hydrogen bonds with His122, His189, Ser217 and Asn220 at the distance of 2.84–3.29 (Fig. 1D). A total of 54 hydrophobic contacts were exhibited by Ile35, Trp93, His122, Gln123, Asp124, His189, Lys214, Ala215, Lys216, Ser217, Gly219 and His250 residues within the range of 2.88–3.90 (Table 2). Highest hydrophobic interactions were exhibited by Lys216 and Ser217, each of which was involved in thirteen and eight contacts, respectively. Whereas, His122 and Gln123 were found to make five hydrophobic interactions.

#### AW01120 (M61)

This compound was found to bind with a GOLD fitness score of 109.17 with lower binding affinity, −8.57 kcal/mol (Table 1). It formed two hydrogen bonds with Lys211 and Asn220 by the distance of 3.00 and 3.16, respectively (Fig. 1E). Oxygen atoms (O8 and O13) of this compound have made hydrogen bonds with these amino acids. A total of 44 hydrophobic interactions were shown by Ile35, Met67, Trp93, His122, Gln123, Asp124, His189, Cys208, Lys211, Ala215, Lys216, Ser217, Gly219, Asn220 and His250 within the range of 3.00–3.87. For NDM-1 no inhibitor is available, thus we have used mercaptocarboxylate (metal enzyme inhibitors) for initial stage comparison.

### Molecular dynamic simulation

**Figure 2:**
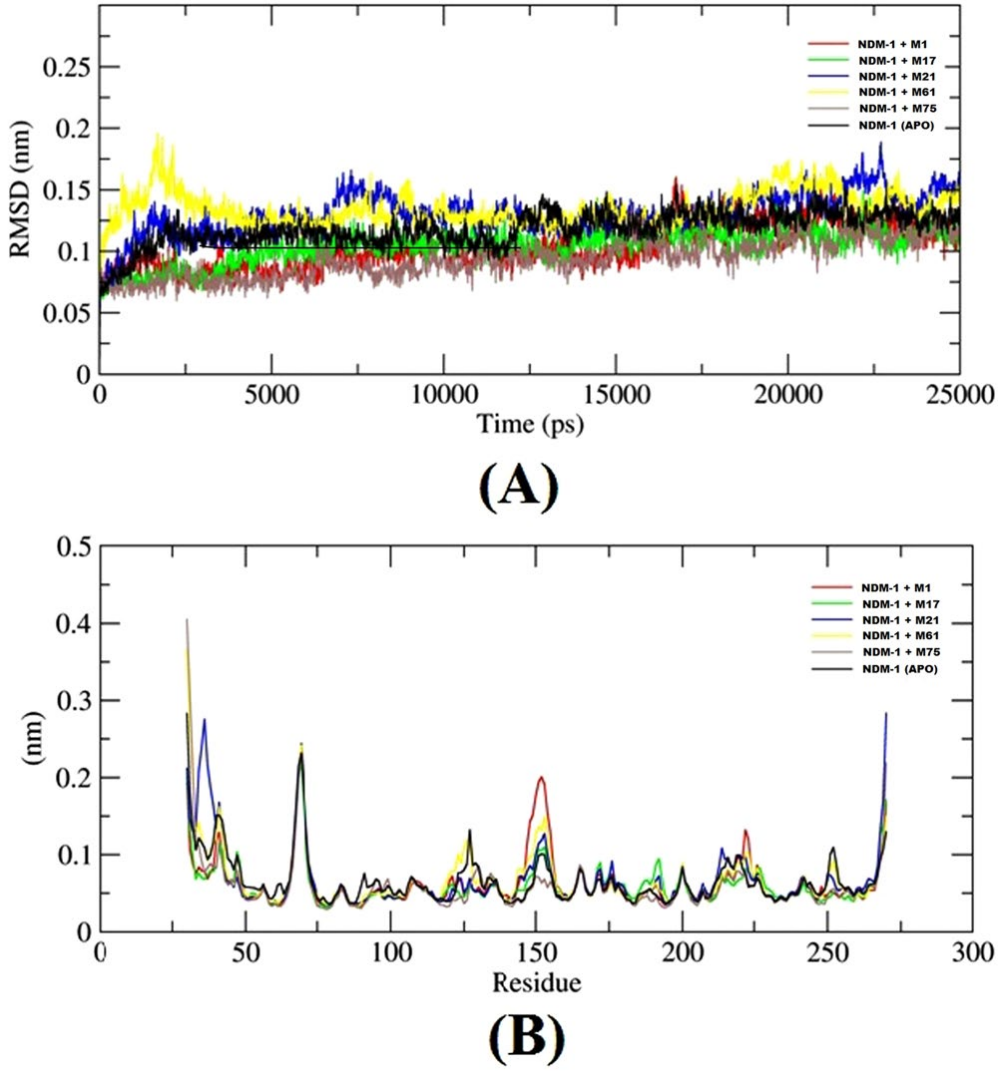
(**A**) RMSD analyses of NDM-1 complexes and (**B**) RMSF analyses of NDM-1 complexes.

To access the stability of the enzyme-ligand complexes, structural drift were monitored with respect to their initial conformations. In this study, 25 ns of molecular dynamics simulation was performed for each complex. Figure 2A shows the RMSD value of the NDM-1-ligands complex structures over the simulation time. RMSD plot has shown relatively stable backbone trajectories of NDM-1 with M75 (average 0.93 Å), M1 (average 1.02 Å) and M17 (average 1.03 Å) as compared to NDM-1 (average 0.93 Å). Whereas, binding of M21, (average 1.29 Å) and M61, (average 1.34 Å) have shown relatively less stable trajectories of NDM-1. Hence, it can be concluded that out of these small molecules, M75, M1, and M17 are the most preferable lead compounds for NDM-1. In the RMSF profile. One high peak was found between the residue 145–155, where it was observed that only M75 restricted its movement while in all other ligand bound system and NDM-1, high fluctuation was observed in this region (Fig. 2B). Our results suggested that enzyme and selected compounds were able to maintain their structural integrity during most of simulations, exhibiting our model had reached a conformational steady state.

H-bond profile of NDM-1 docked systems showed consistent H-bond trajectory in M75 and M1, docked with NDM-1, where average 2 h-bond were found throughout the trajectory. In the M17 docked system, only one h-bond was found throughout the time.

On the other hand, among all the five screened compounds, compound 61 has shown highest binding affinity against NDM-1 with binding energy of −20.129 kJ/mol (Table 3).

**Table 3:** Binding free energy of selected ligands against NDM-1 protein.

NDM-1					
	M1	M17	M21	M61	M75
van der Waal energy	−110.901 +/− 13.135	−149.851 +/− 10.089	−139.477 +/− 11.860	−188.133 +/− 10.343	−11.158 +/− 27.816
Electrostatic energy	74.576 +/− 16.843	83.009 +/− 8.351	30.664 +/− 12.140	26.428 +/− 13.435	−1.889 +/− 6.720
Polar solvation energy	143.227 +/− 31.952	123.378 +/− 20.348	164.221 +/− 19.106	163.307 +/− 22.350	44.225 +/− 47.823
SASA energy	−17.983 +/− 1.400	−19.377 +/−1.044	−19.365 +/− 1.373	−21.730 +/− 0.840	−1.197 +/− 3.480
Binding energy	88.920 +/− 44.875	37.159 +/−19.201	36.043 +/− 20.291	−20.129 +/− 16.452	29.982 +/− 47.322

### Microbiological analysis

The *bla*_NDM-1_ gene was cloned in pQE-2 vector and transformed into *E. coli* DH5α cells, confirmed by PCR (Figure S3). These ideal *bla*_NDM-1_ harbouring clones were used to check the inhibitory potential of designed inhibitors. The MIC was determined for *E. coli* DH5α cells, *E. coli* DH5α cells with vector and *E. coli* DH5α cells with *bla*_NDM-1_ carrying vectors. In Table 4, we have summarized the MICs of antibiotics alone and with inhibitors combination for NDM-1 carrying clones. The MICs of the β-lactam antibiotics used in this study were higher for NDM-1 expressing clones. We next assayed the activities of these drugs in combination with novel screened molecules. For *bla*_NDM-1_, the lower MICs were obtained when each of the five molecules (AW01220 (M1), BTB02323(M17), RF01991(M21), AW01120 (M61) and HAN00044 (M75) were combined with the ceftazidime, cefoxitin, meropenem and imipenem. The MICs were lowered from 8 to 4 µg/ml (ceftazidime), 16 to 8 µg/ml (cefoxitin) and 2 to 1 µg/ml for meropenem and imipenem (Table 4).

**Table 4:** Minimum inhibitory concentration values (µg/ml) of antibiotics and antibiotic-inhibitor combinations for *E. coli* DH5α transformed with recombinant NDM-1 and original DH5α (with null vector).

Compound	(pQE-2- NDM-1) DH5α	(pQE-2- Original) DH5α
Cephotaxime	512	0.25
Cephotaxime + BTB02323	256	0.25
Cephotaxime + RF01991	256	0.25
Cephotaxime + AW01220	256	0.25
Cephotaxime + HAN00094	256	0.25
Cephotaxime + AW01120	256	0.25
Ceftazidime	8.00	0.25
Ceftazidime + BTB02323	4.00	0.25
Ceftazidime + RF01991	4.00	0.25
Ceftazidime + AW01220	4.00	0.25
Ceftazidime + HAN00094	4.00	0.25
Ceftazidime + AW01120	4.00	0.25
Cefoxitin	16.0	2.00
Cefoxitin + BTB02323	8.00	2.00
Cefoxitin + RF01991	8.00	2.00
Cefoxitin + AW01220	8.00	2.00
Cefoxitin + HAN00094	8.00	2.00
Cefoxitin + AW01120	8.00	2.00
Meropenem	2.00	0.50
Meropenem + BTB02323	1.00	0.50
Meropenem + RF01991	1.00	0.50
Meropenem + AW01220	1.00	0.50
Meropenem + HAN00094	1.00	0.50
Meropenem + AW01120	1.00	0.50
Imipenem	2.00	0.50
Imipenem + BTB02323	1.00	0.50
Imipenem + RF01991	1.00	0.50
Imipenem + AW01220	1.00	0.50
Imipenem + HAN00094	1.00	0.50
Imipenem + AW01120	1.00	0.50

### Determined IC50 Value

**Figure 3:**
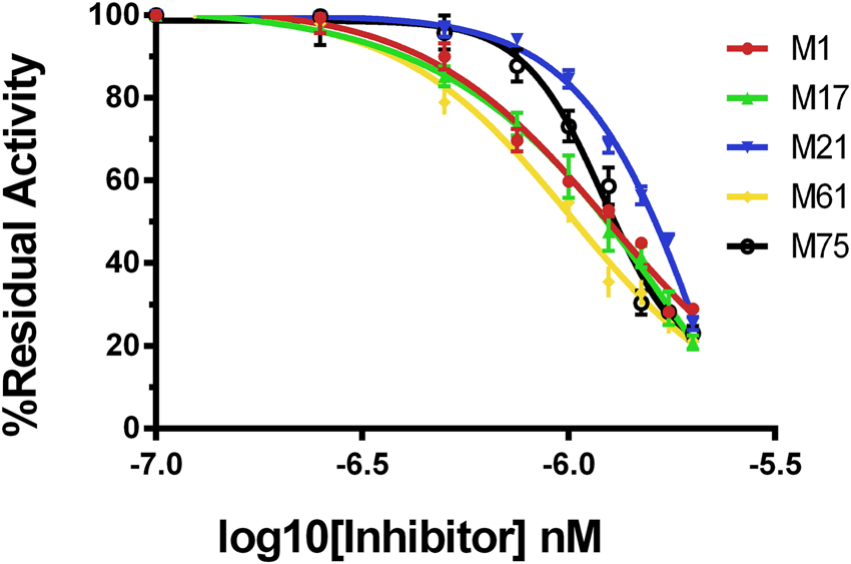
Representation of IC50 values for various type of inhibitors with residual activity of NDM-1as monitored by the hydrolysis of different concentration of inhibitor with constant value of 100 μM nitrocefin.

The IC50 values were properly calculated to know the potency of each inhibitor and compared their efficacies under properly controlled experiments. The IC50 values were calculated by appropriate protein NDM-1 with each of its inhibitor (M1, M17, M21, M61 and M75) at fixed concentration of nitrocefin substrate (100μΜ) (Table 5). IC50 values for NDM-1 inhibitors were calculated as M1 (1.244 nM), M17 (1.904 nM), M21(2.283 nM), M61(1.009 nM) and M75 (1.21 nM) as shown in Fig. 3. Which were found comparable to known NDM-1 inhibitor, EDTA (0.25 nM) and D-captopril (7.9 nM)^9^.

**Table 5:** Inhibitor concentration required to reduced 50% enzyme activity.

IC50 (nM) values				
NDM-1				
M1	M17	M21	M61	M75
1.244	1.904	2.283	1.009	1.212

### Analysis of the steady state kinetics parameter

**Figure 4:**
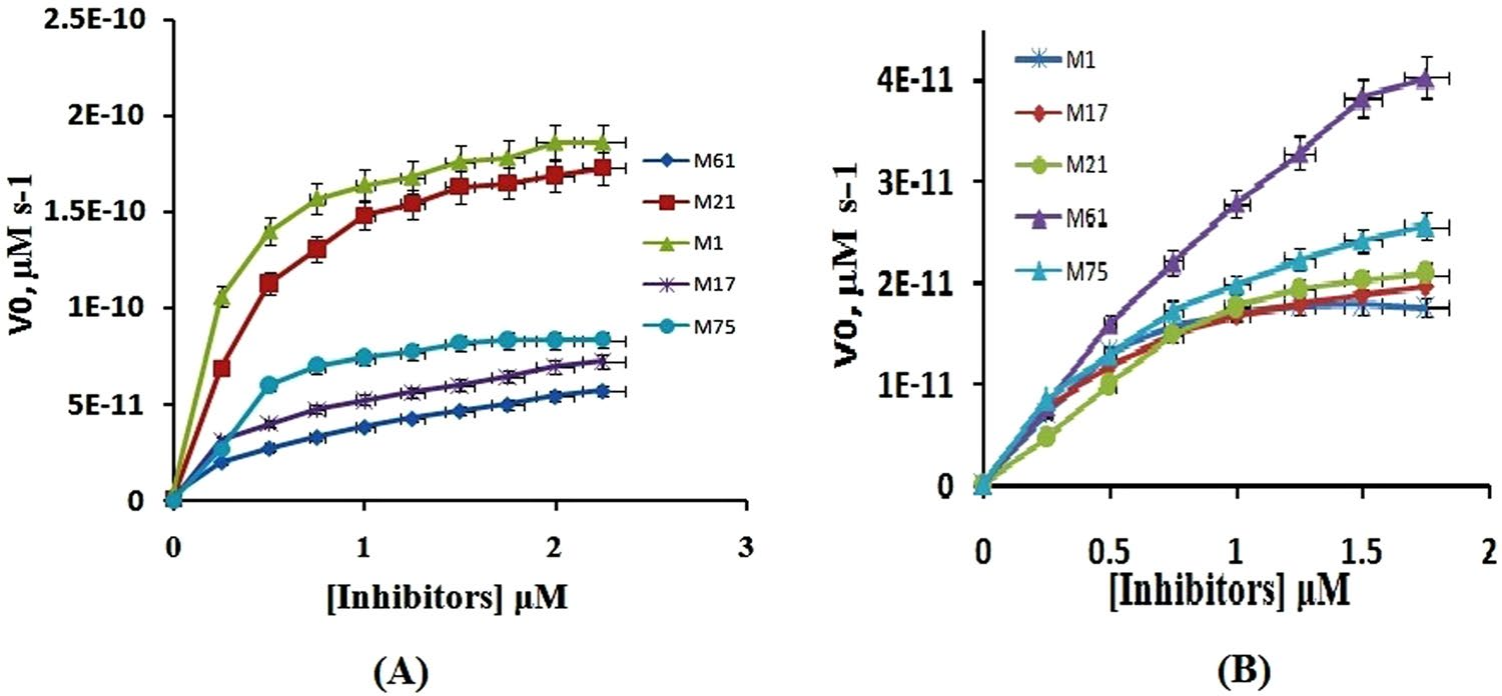
Determination of different types of inhibitor and different antibiotics for the NDM-1, represented the best fit (non linear regression). Panel 4 A shows inhibitor M1, M17, M21, M61 and M75 with antibiotics Meropenem. Panel 4B shows inhibitor M1, M17, M21, M61 and M75 with antibiotics Imipenem.

The enzyme kinetics parameter, Km, Vmax and Kcat values for purified protein NDM-1 and each of these antibiotics (meropenem and imipenem), were calculated. The inhibitor of the NDM-1(M1, M17, M21, M61, M75) were carried out as per the concentration of IC50 value in the reaction, represented by Michaelis-menten equation (Fig. 4A,B) whereas, the kinetic parameters are summarized in Table 6.

**Table 6:** Enzyme kinetics parameter for NDM-1 protein with substrate (Antibiotics with Inhibitor).

Protein	NDM-1									
Antibiotics	Meropenem					Imipenem				
Inhibitors	M1	M17	M21	M61	M75	M1	M17	M21	M61	M75
Km or Ki (μΜ)	44.76	23.49	52.03	98.29	64.20	42.90	61.89	126.8	304.3	99.98
Kcat (S^−1^)	208.6	204.5	110.8	791.2	901.8	233.5	269.8	378.9	112.8	403.3
Kcat/Km (μM^−1^s^−1^)	4.660	8.705	2.129	8.049	14.04	2.335	4.359	2.988	0.3700	4.037

Kinetics parameters of NDM-1 represented the hydrolytic profile that is a feature of molecular class A and class B type β-lactamases, respectively. The enzyme kinetics parameter showed good affinity and catalytic activity with all the antibiotics, imipenem and meropenem (Km in 20 to 200 μM range). Different drugs in combination with different inhibitors showed varying affinity with NDM-1 as shown in Table 6.

### Fluorescence mechanism and binding affinity of NDM-1 with their inhibitors

Fluorescence quenching can be either dynamic or static in nature. To understand the quenching mechanism of NDM-1 with M1, M17, M21, M61, M75, the fluorescence intensity was calculated and was found gradually decreased along with 1 nm blue shift due to quenching of NDM-1 fluorescence as shown in Figure S4. We determined the binding constant (Kb) and the number of binding sites (n) and Stern-Volmer quenching constant (Ksv) for NDM-1 protein with M1, M17, M21, M61, M75, respectively at 298 K temperature by using the following Stern-Volmer equation and modified Stern-Volmer equation 1 and 2.

1 $$({\rm{F0}})/{\rm{F}}=1+{\rm{Ksv}}[{\rm{Q}}]$$

2 $$\mathrm{Log}(F0-F)/{\rm{F}}=\,\mathrm{log}\,{\rm{Ka}}+{\rm{n}}\,\mathrm{log}[{\rm{Q}}]$$

**Figure 5:**
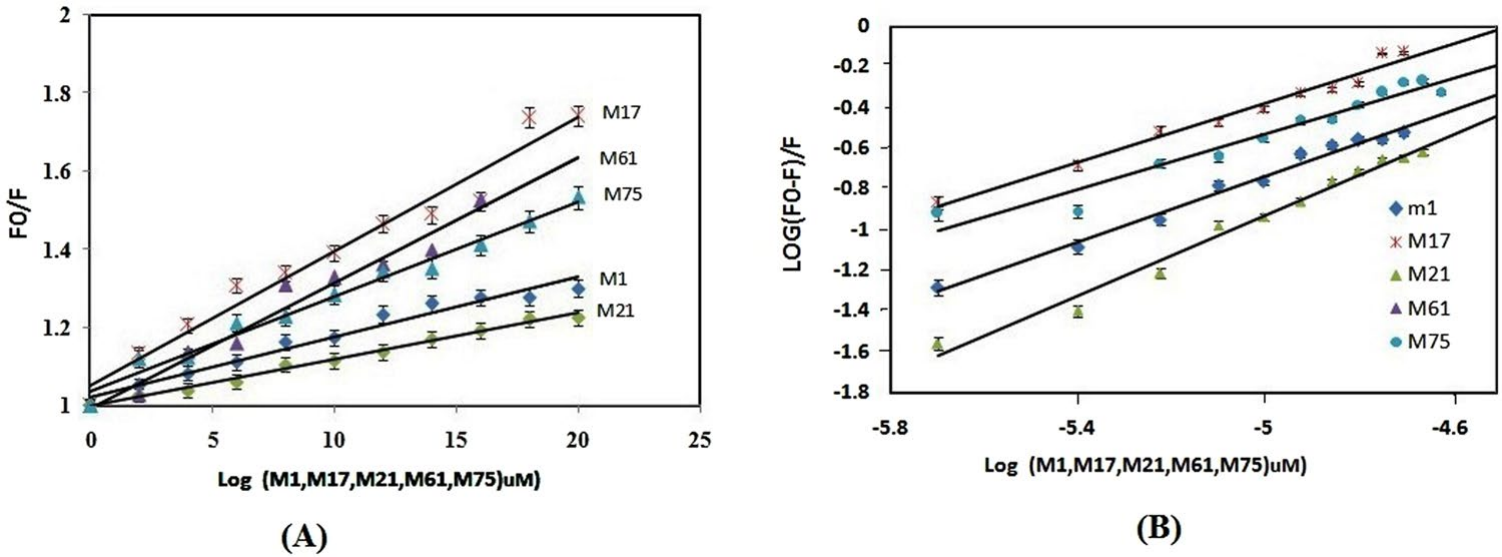
(**A**). M1, M17, M21, M61, M75 -induced fluorescence quenching of NDM-1 at 298 K. (**A**) Stern−Volmer plot and (**B**) modified stern Volmer for NDM-1−M1, M17, M21, M61,M75 interactions. The concentration of NDM-1 was 2 μM in 20 mM sodium phosphate buffer at pH 7.0.

Where Fo and F are the fluorescence intensities in the absence and presence of quencher M1, M17, M21, M61, M75. Ksv were calculated from the slope of plot Fo/F vs [Q], whereas, the slope and intercept of plot log (Fo/F − 1) vs log[Q] determined n and Kb values as shown in Table 7. The values for binding constant and K_SV_ were shown in Fig. 5A,B and separate figure shown in supplementary material section (Figures S5 to S7).

**Table 7:** Binding parameters obtained from Fluorescence Quenching Experiments.

Protein	NDM-1				
Inhibitors	M1	M17	M21	M61	M75
K_SV_ × (M−1)	1.50 × 10^4^	3.40 × 10^4^	1.10 × 10^4^	3.29 × 10^3^	2.10 × 10^3^
Kb (M−1)	1.90 × 10^3^	1.60 × 10^3^	9.70 × 10^3^	9.60 × 10^5^	7.30 × 10^2^
n	0.805	0.723	0.984	1.298	0.679
R^2^	0.966	0.966	0.989	0.968	0.948

In this study Ksv were obtained, as in the order of 10^4^ and 10^3^ (Table 7). Result showed that quenching is not initiated by dynamic diffusion but occurred by formation of a strong complex between NDM-1 with their inhibitors (M61, M21, M1, M17, M75), in decreasing order. M61 was found to form a strong complex with NDM-1.

### Isothermal titration calorimetric (ITC) measurements

**Figure 6:**
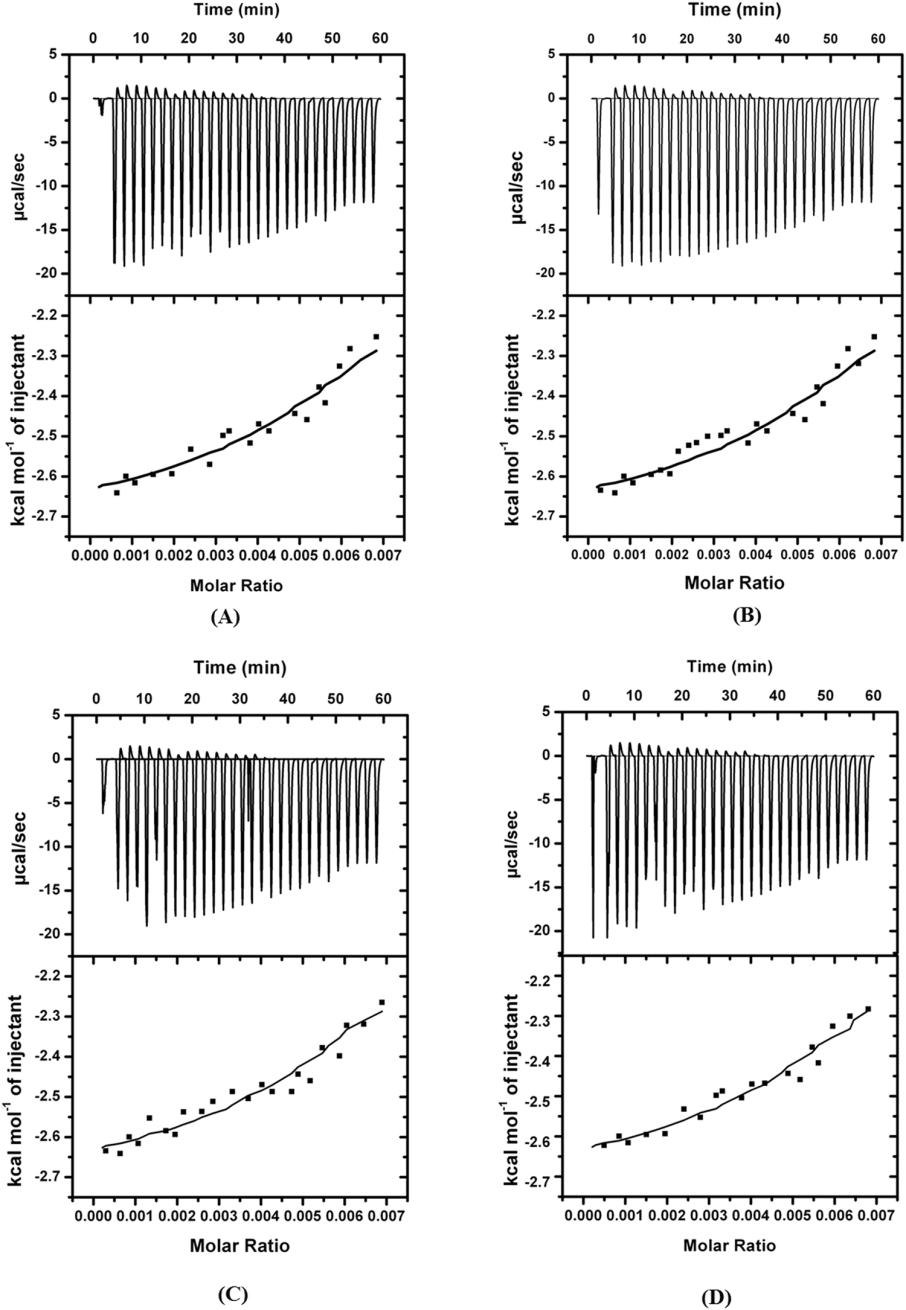
Isothermal titration calorimetry of NDM-1 protein with M1, M17, M21, M61, shown in panel as (**A**,**B**,**C** and **D**), respectively at 298 k temp, represent ITC profile.

ITC allows the measurement of binding affinity magnitude, and the two contributing thermodynamic terms, enthalpy (ΔH) and entropy (ΔS) changes. A representative calorimetric titration profile of the M61with NDM-1 at 25 °C is shown in Fig. 6. In the top panel; each peak represents a single injection of the inhibitor into protein solution. The bottom panel shows an integrated plot of the amount of heat liberated per injection as a function of the molar ratio of the inhibitor to protein. ITC is a quantitative technique was used to determined enthalpy change (∆H), binding affinity (Ka) and binding stoichiometry (n) of the interaction between two or more molecule of the solution. From there initially measurement of entropy changes (ΔS) and Gibbs energy changes (ΔG) were calculated using equation 3.

3 $${\rm{\Delta }}{\rm{G}}={\rm{\Delta }}{\rm{H}}-{\rm{T}}\times {\rm{\Delta }}{\rm{S}}$$

The titration of M61, M21, M17, M1 with NDM-1 were performed. Negative heat deflection at all studied temperatures were observed, indicating that the binding is an exothermic process (Fig. 6). We fit the data for single sites by the sequential binding model. The values of binding constant obtained are of the order of 10^4^ to 10^6^ with each inhibitor and thus can be referred as high and low affinity sites, respectively, at studied temperature. Result of the binding of inhibitors with protein is shown in Table 8. As evident from the value of ΔH and ΔS, the binding becomes more exothermic.

**Table 8:** Thermodynamic Parameters for the Binding of NDM-1 protein with M61, M21, M17, M1 respectively as Estimated by Isothermal Titration Calorimetry.

	Kb (M−1)	Kd (mM)	N (Site)	ΔH(kJmol−1)	TΔS (kJmol−1)	ΔG (kJmol−1)
NDM-1,M61	7.640 × 10^4^	1.303	0.0656	−29.30	−1.832	−27.46
NDM-1,M21	5.310 × 10^4^	1.884	0.0546	−27.30	−1.677	−25.62
NDM-1,M17	5.000 × 10^4^	2.000	0.0536	−25.30	−1.609	−23.69
NDM-1,M1	4.930 × 10^4^	2.028	0.5060	−21.30	−1.434	−18.64

### Haemolysis assay and MTT

We had performed RBC lysis test and mitochondrial activity based toxicity assay, MTT. The pre-incubation of our molecules with healthy cells showed very little toxicity and causes limited lysis of cells. As shown in Figure S8, compound (M61) caused lysis by 8.2%, even at a high concentration (50 μg/ml). However, as the concentration increased the cell viability decreased proportionally.

The MTT assay was carried out on PBMCs and the results demonstrated a concentration and time dependent cytotoxicity after exposure to compounds. The surprising upshot of study was the observation that our compounds failed to show cytotoxic effects on PBMCs, even at the 5 times of the MIC concentration (~100 μg/ml) and showed remarkable (82.3% and 82%) cell viability (P ≤ 0.001). However, treatment with increasing doses of compounds reduced the cell viability.

## Discussion

The worldwide dissemination of NDM-1 has become a major threat to human health. For metallo-β-lactamase, no inhibitors are available. This limitation encouraged us to design a novel class of β-lactamases and metallo-β-lactamase inhibitors. The identification of novel non-covalent inhibitors of β-lactamases is a promising approach to maintain the effectiveness of β-lactam antibiotics. The purpose of this initial study was to identify hotspots of the active site that could be more productively engaged with designed lead molecules against NDM-1type β-lactamases. From our docking analyses, amino acid Ser237 was found crucial in stabilizing the complex by hydrogen bonds or hydrophobic interactions. In this study, we have cloned *bla*_NDM-1_. Antimicrobial activity was checked quantitatively to determine the minimum inhibitory concentrations (MICs) of the antibiotic-inhibitor combination necessary to inhibit bacterial growth, imipenem and meropenem, these compounds for NDM-1showed lowered MIC values (Table 4).

In case of NDM-1, His122, His189, Cys208, Lys211, Asn220 and His250 have been observed to interact with compounds by hydrogen or hydrophobic interactions. These amino acids are crucial for substrate recognition and found to be conserved in metallo-β-lactamases^10^ all the five compounds tested were found to interact with these amino acids either by hydrogen bond or hydrophobic interactions. Most stable complexes were found, are M21, M1 and M75. Another compound, M61 formed five hydrogen bonds and seventy hydrophobic interactions with the crucial amino acids His122, Asp124, Cys208, His189 and His250. Active site of NDM-1 have two zinc ions (Zn1 and Zn2 site), where Zn1 coordinated by three histidine (His120, His122, and His189) residues, and second zinc ion (Zn2) is coordinated by Asp124, Cys208, and His250^11^. Our study showed that all the molecules have hydrophobic interactions with Asp124. Amino acid, Asn220 was found to be crucial for hydrogen bonding. This result is in agreement with the study which showed that carbonyl group of ampicillin was hydrolyzed to a carboxylate group by forming a hydrogen bond with residue Asn220 which is a conserved residue in subclass B1 and B2 MBLs^9^. Despite these, Gln123, Ala215, Lys216, Ser217 and Gly219 play an important role in stabilizing the complex through hydrophobic interactions. Among these, Gly219 is known to be conserved in subclass B1 MBLs, which are proposed to participate in the catalysis reaction^12–14^. All the five compounds selected for NDM-1 are in the range of Lipinski’s rule of 5. Moreover, these compounds were also found stable through the molecular dynamic simulation. Many previous studies have already proved that the combination of the antibiotic with different type β-lactam based inhibitor may be an effective approach to control word wide dissemination of the β-lactamases^15^. In this study, β-lactamase inhibitors for class B were studied using MICs values of different β-lactam antibiotics with their different combination of each inhibitor^7^. These β-lactamases showed the hydrolysis with their antibiotics by two step mechanism, acylation and deacylation method, where hydroxide is bound to the acyl carbonyl and hydrolyzes the acyl enzyme intermediate^16^. Deacylation rate is very high at the time of interaction with β-lactam antibiotics and the active enzyme is quickly regenerated. Whereas, on the other hand significant stability of acyl-enzyme complex is made due to interaction of β-lactam inhibitors.

In this study, we observed that each of the inhibitors was found to have a great affinity with their specific target enzyme (NDM-1). The recombinant protein NDM-1 was used to understand the binding affinity and efficiencies with M1, M17, M21, M61, M75, respectively, using fluorescence spectroscopy. The mechanism of fluorescence is observed as the result of photon emission when an electron in a higher energy level is returned back to a lower energy level^17^. In this process, quenching is observed due to various molecular interaction such as reactions in the exited state, molecular interaction rearrangement, energy transfer and static and dynamic quenching. The measurement of intrinsic fluorescence quenching of protein has been extensively used to understand a mechanism of enzyme inhibitor interaction^18^. Two types of quenching mechanisms are known, dynamic and static. In case of static quenching it forms a ground state complex with the molecule while in dynamic quenching, the quencher indirectly interacts with the molecule^19^. The blue and red shift indicate the increase or decrease in hydrophobicity around Trp and Tyr residues, respectively^20^. The measurement of intrinsic fluorescence quenching of protein has been widely used to elucidate the mechanism of its interaction with a drug molecule^18^.

The binding affinity of M61, M21, M17 and M1 to NDM-1 and associated thermodynamic parameters were further determined by ITC. It is a sensitive technique to determine micro environmental and conformational alteration associated thermodynamic parameters induced by ligand interaction. Thermodynamic properties such as change in enthalpy (Δ*H*°) and entropy (Δ*S*°) were determined for NDM-1 and the representative calorimetric titration profile with lowest χ2 value after best fitting by “single binding site model is shown in Fig. 6A,B,C,D. The enthalpy (ΔH) was determined directly from the isotherm and is the amount of heat released per mole of ligand bound. Hence, the single ITC experiment delivers prosperous information about the binding reaction which is very useful to understand the nature of the interaction and exploring the thermodynamic mechanism. In the upper panels of the Fig. 6A,B,C,D. multiple single injection of the each inhibitor into protein solution was shown as peak of the binding isotherm. While, the lower panel shows integrated plots of heat released per injection as a function of protein-inhibitor molar ratio^21^. The binding of each inhibitor (M61, M21, M17, M1) to NDM-1 shows an exothermic pattern and the related Gibbs free energy and entropy changes. The signal again returns to its starting position, when the temperature of the two cells are equal. The results of the fluorescence spectroscopy and ITC were in agreement with that of fluorescence spectroscopy. However, variation in magnitude of binding affinity and thermodynamic parameters were observed, which may be due to the fact that the calorimetric analysis measures overall change in the property of a system, whereas, spectroscopic analysis measures narrow changes around the chromophores / fluorophores associated with the optical transition. The binding stoichiometry obtained from calorimetry and spectroscopy was found slightly conflicting which might be due to the photo physical problem because of the influence of Trp in NDM-1^17, 19^. Enthalpies of binding have conflict with buffer ionization because phosphate and other buffer content have a little value of the enthalpy (3.6 kJ mol^−1^)^22^, the observed enthalpy values are basically the binding enthalpies of the inhibitors and protein^23^. This study introduces an efficient virtual screening protocol to discover the novel non β-lactam inhibitors against the NDM-1 type β-lactamases. It was accomplished by virtual screening for inhibitors and their experimental evaluation. This multi-step procedure resulted in three compounds for NDM-1 that was experimentally confirmed as potent inhibitors. Based on the docking energy scores and extensive experimental evaluation, it was found that compounds, M1, M17, M21 and M61 showed high affinity against the NDM-1. Hence these molecules may be proposed as potential leads to design future drug candidates.

## Experimental Procedures

### Enzyme and ligand preparation

Crystal structures of NDM-1 (PDB code: 3Q6X), IMP-1 (PDB ID: 3WXC) was downloaded from RCSB Protein Data Bank (http://www.rcsb.org/pdb). All water molecules (except catalytic) were removed and hydrogen atoms were added to enzyme using Discovery Studio 2.5 (DS, Accelrys Inc, San Diego). The binding sites were defined based on the residues 10 Å around the crystal structure of known ligand position in complex with the target. Potential steric clashes and added hydrogen atoms were relaxed by using the minimization procedure. Energy minimization was performed by using the simulation module of the DS 2.5 with conjugate gradient method^24^ after assigning the CHARMm force field^25^. This process was carried out until the average absolute derivative of coordinates with respect to energy fell below the 0.1 cal Å−1.

### Chemical Library

Virtual screening was performed to identify possible hits compounds from the Maybridge HitFinder™ database. The Maybridge HitFinder™ sets are structural representatives of large non-redundant chemical libraries. This collection includes ~58,000 compounds that represent the drug-like diversity of the Maybridge Screening Collection. All the screening compounds fit Lipinski guidelines for drug-likeness; partition coefficient, ClogP ≤ 5, H-bond donors ≤ 5, H-bond acceptors ≤ 10, molecular weight 500. The Maybridge HitFinder™ set was obtained from http://www.maybridge.com website. Tanimoto Coefficient of similarity for molecules A and B by using the equation 4.

4 $$T.C=\frac{C}{A+B-C}$$

Where C is number of common “1” bits that occurs in both active and inactive molecules; A is number of “1” bits in fingerprint active molecules; B is number of “1” bits in fingerprint inactive molecules.

### Molecular docking

The number of programs available to assess and rationalize ligand protein interactions are many and ever increasing. Revolution in computational speed provides a plethora of techniques to tackle modern structure based drug design problems. In this work we have used GOLD and AutoDock Vina to predict the conformation of a receptor-ligand complex.

GOLD 5.0 version^26^ was used for virtual screening of the compound dataset. Docking annealing parameters for Van der Walls and hydrogen bonding were set to 5.0 and 2.5 respectively. The parameters used for genetic algorithm were; population size 100, selection pressure 1.2, number of operations 1,00,000, number of islands five, niche size 2, migrate 10, mutate 100 and cross-over 100. From each docking solution, conformation with best fitness score was extracted. A simulation box of 40 × 40 × 40 A° was used in each docking calculation with an exhaustiveness option of 8. The pdbqt files of proteins and ligand were prepared using AutoDock Tools 4.2.6^27^. Polar hydrogen was added and the binding free energies were calculated using scoring function of AutoDock Vina.

### Post-docking analyses and molecular dynamic simulation

X-Score, a consensus scoring function^28^, was used in order to carry out docking validation. It uses the negative logarithm of the dissociation constant of the ligand to the protein, −log Kd, as the average of three scoring functions (HPScore, HMScore and HSScore). Profiles of interacting amino acid residue pairs were obtained by using the Ligand interaction script in Accelrys® Discovery Studio visualizer and Ligplot program^29^. These programs calculate the number of hydrogen bonds and non-bonded contacts between the target and chemical structure. Molecular dynamic simulation was performed to check the stability of ligand within the active site of the enzyme for 25 ns time interval. Gromacs 5.1^30, 31^ was used for MD simulation studies. Topology files of macromolecules and small molecules were prepared by using Amber99sb-ildn force field and ANTECHAMBER module of AMBER Tools, respectively^32, 33^. Apo or docked system was placed in the centre of cubic box having distance of 10.0 A° between protein and edge of the simulation box and solvated with TIP3P^34^ explicit water molecules. Systems were neutralized by adding 3 CL and 2 NA ions in NDM-1 bound systems, respectively. Each system was minimized by using steepest descent approach for 1 ns. Further NVT and NPT were also performed for 1 ns to equilibrate the systems for constant volume, pressure (1 atm) and temperature (300 K). Trajectories were further generated for analysis, using Xmgrace^35, 36^.

### Binding energy calculation

Binding free energy calculations for all the docked complexes were performed by Molecular Mechanics/Poisson-Boltzmann Surface Area (MM/PBSA) method of g_mmpbsa module^37, 38^. Total 500 snapshots were taken from the last 5 ns trajectory. g_mmpbsa module calculates electrostatic interactions, Van der Waals interactions, polar salvation energy and non-polar solvation energy.

### Antimicrobial agents

Antimicrobial agents used in this study were: ampicillin, Imipenem, meropenem, cefoxitin, ceftazidime, were purchased from sigma, USA. Whereas, novel scaffold inhibitors (M1, M17, M21, M61 and M75) used in this study were purchased from Scitech Scientifics.

### Bacterial strains and culture conditions

*E. coli* DH5α and *E. coli* BL21 (DE3) were used for cloning. The clinical strain of *Enterobacter cloacae* (EC15) was obtained from lab stock^39^. The strains was routinely grown over night in Luria Bertani (LB) broth at 37 ^ο^C and maintained as glycerol stock in −80 °C.

### Cloning and expression

The plasmid DNA harbouring The *bla*_NDM-1_ gene was amplified by PCR with the primers NDM-1F (5′ ATATCATATGGAATTGCCCAATATT 3′) containing Nde I site and NDM-1-R (5′ ATATAAGCTTTCAGCGCAGCTTGTCGG-3′) containing Hind III site. The PCR conditions used, were 95 °C, 7 minute, 95 ^ο^C, 30 s, 55 ^ο^C, 30 s, 72 ^ο^C 1 minutes, and the reaction was carried out for 35 cycles. The PCR product does not contain the promoter region of the gene. The PCR product was purified using Gene Jet Gel Extraction Kit according to the instructions manual. The process of digestion and ligation were carried out according to the protocol provided by the enzyme manufacturer with some modification (Fermentas). The PCR product and pQE-2 (high copy expression vector), were double digested with Nde I and Hind III (Fermentas FastDigest) and incubated at 37 ^ο^C for an hour. The digested product was confirmed on agarose gel (0.8%) and again purified before proceeding to ligation. The enzyme T4 ligase (Fermentas T4 ligase) was added to digested product and incubated at 16 ^ο^C for overnight. Further, the enzyme ligase was deactivated by incubating at 65 ^ο^C for ten minutes and the ligated product was used to transform competent *E. coli* DH5α by heat shock method. Transformants, harbouring NDM-1 gene were selected on LB agar plates containing ampicillin (100 μg/ml).

### Determination of minimum inhibitory concentration

The MICs of four antibiotics were calculated (Table 4). The results were interpreted according to Clinical Laboratory Standards Institute (CLSI) guidelines. *E. coli* cells were treated with increasing concentrations of the antibiotics ranging from 0 to 512 µg/ml in a series of two fold dilutions.

### Determination of inhibition constant (Ki) and IC50 value

IC50 and Ki value were determined by the direct competition between beta-lactamase substrate, nitrocefin and their inhibitors under appropriately controlled experiments. Different concentrations of inhibitor M1, M17, M21, M61 and M75 (0 to 3 μM), fixed concentration of purified protein of NDM-1(1 nM) respectively, and nitrocefin substrate (100 μM) were used in reaction. The rate of hydrolysis of nitrocefin was monitored by the change in absorbance due to cleavage of β-lactam ring at 486 nm using Shimadzu UV-VIS Spectrophotometer UV-1800. The IC50 values were obtained by plotting percent residual enzyme activity on nitrocefin (%) versus inhibitor concentration (log_10_). The 50% inhibitory concentration (IC50) were defined as the concentration of the inhibitor that inhibited hydrolytic activity of the enzyme by 50%. The inhibition constant, Ki, was calculated from IC50 value by applying the Cheng-Prusoff correction by equation 5 ^40^.

5 $${\boldsymbol{Ki}}={\boldsymbol{Ic}}{\bf{50}}/({\bf{1}}+{\boldsymbol{S}}/{\boldsymbol{Km}}\,{\boldsymbol{NCF}})$$

Where Km and [S] corresponds to Michaelis-Menten constant and concentration of nitrocefin respectively.

### Steady state and chemical kinetics parameter (Km, Vmax and Kcat)

The determination of steady-state kinetics parameter of NDM-1 protein with the following antibiotics: and imipenem (Δε295 = −11,500 M^−1^ cm^−1^), meropenem (Δε297 = −10,940 M^−1^ cm^−1^) respectively, was performed. M1, M17, M21, M61 and M75 inhibitors were used in the study. Hydrolysis of β-lactam antibiotics were detected by monitoring the variation in the absorbance due to cleavage of β-lactam ring in 50 mM phosphate buffer, pH 7.0^41^. All the measurements were taken in triplicate on Shimadzu UV-VIS spectrophotometer (UV-1800). The reaction was performed in a total volume of 1000 μl at 30 °C. BSA was added 20 μg/ml for dilution of the enzyme and prevent the reaction denaturation.

For hydrolysis of substrates (Imipenem and meropenem) kinitics parameter Km, Vmax and Kcat were calculated by using Michaelis-Menten equations 6 and 7.

6 $$V=\frac{{\boldsymbol{Vmax}}}{{\boldsymbol{Km}}}\frac{[{\boldsymbol{S}}]}{[{\boldsymbol{s}}]}$$

7 $${\boldsymbol{Kcat}}\,=\,\frac{{\boldsymbol{Vmax}}}{{\boldsymbol{E}}}$$

Where, Vmax and V are the maximam and initial velocity of the hydrolysis respectively, Enzyme concentration [E], Substrate concentration [S] and Michaelis-Mentent constant is Km. Kcat values were determined from the initial rates calculated at saturating substrate concentration (as a zero^th^ order reaction of kinetics), and Km value were determined as competitive inhibition constant (Ki) in a competition experiment between tested antibiotic/inhibitor and 100 mM nitrocefin used as reporter substrate, and the result was analysed according to equation 8 ^31^.

8 $$\frac{{\boldsymbol{V}}0}{{\boldsymbol{Vi}}}=1+(\frac{[{\boldsymbol{KmC}}]}{{\boldsymbol{Km}}+[{\boldsymbol{S}}]}).{\boldsymbol{Ki}}\,$$

Where Vi and Vo represent the initial rate of substrate hydrolysis in the presence and absence of the substrate, respectively, [C] is the concentration of the substrate/inhibitor, [S] and Km are the concentration of substrate and Michaelis-Menten constant, respectively.

### Fluorescence spectra measurements

Fluorescence spectra were measured on a Shimadzu RF-5301PC spectrofluorometer (Shimadzu Corporation, Kyoto, Japan) equipped with a thermo statically controlled cell holder and attached to a water bath. Fluorescence measurements can give some information on the binding of small molecules to the protein, such as the binding mechanism, binding mode, binding constants, binding sites and intermolecular distances. Quenching was monitored by measuring intrinsic fluorescence between 300 and 400 nm at 295 nm. Both the excitation and emission slits were set at 5 nm. 3 mL sample containing 2 μM NDM-1 protein was successively added 2 μM of each M1, M17, M21, M61, M75 respectively in such a manner that the total volume added was not more than 30 μL. All the fluorescence intensities were corrected for the inner filter effect.

### Thermodynamics and binding parameters by ITC

ITC measurements were performed on ITC-200 microcalorimeter (MicroCal Inc., Northampton, MA) at 298 K. Protein samples, NDM-1 (15 μM) and inhibitors (M61, M21, M17, M1,) (1 mM) and 50 mM sodium phosphate buffer (pH 7.0) were properly degassed by using ThermoVac unit before performing experiments. Multiple injections of M61, M21, M17, M1C1 solutions were made into the sample cell containing NDM-1 respectively. Each injection was made over 20 s with an interval of 180 s between successive injections. The reference power and stirring speed were set at 16 μcal s^−1^ and 307 X g, respectively. Heat of dilution for the ligand was determined in the control experiment and was subtracted from the integrated data before curve fitting. The data were fitted and analyzed according to the sequential binding with single binding sites using Origin 7.0 software, provided with the instrument.

### *In vitro* erythrocyte lysis test

It was carried out as a preliminary toxicity test of these inhibitors, which is assessed by measuring the haemoglobin released as a result of membrane leakage or disruption caused by exposure to low doses of these molecules. Briefly, fresh blood obtained from a healthy rabbit was collected in anticoagulant solution (EDTA) and centrifuged at 1000 g for 10 min at 4 C. Both buffy coat and plasma were discarded. Washed erythrocytes were diluted with isotonic buffer (20 mM PBS) to prepare 50% haematocrit. Extent of haemolysis was studied by incubating the RBC suspension with various molecules at a different concentration at 37 °C for 1 h. The incubated solutions were centrifuged at 1500Xg for 15 minutes and supernatant was collected and analysed by ultraviolet-visible spectroscopy ($\lambdabar$ max = 576 nm) for released haemoglobin. The percentage haemolysis was determined by the following equation 9:

9 $$ \% \,{\rm{Haemolysis}}=\{({\rm{Abs}}({\rm{T}})-{\rm{Abs}}({\rm{C}})/{\rm{Abs}}(100 \% )-{\rm{Abs}}({\rm{C}})\}\ast 100$$

where Abs(T) is the absorbance of the supernatant from samples incubated with the particles, Abs(c) is the absorbance of the supernatant from controls (normal saline), and Abs(100%) is the absorbance of the supernatant of controls incubated in the presence of 1% Triton® X-100, which causes complete lysis of RBCs (total lysis).

### MTT Assay on PBMCs

Peripheral blood monocyte cells (PBMCs) were isolated from human blood using ficoll reagent. PBMCs (1 × 10^5^ cells/well) were grown in 96-well plates at 37 °C, 5% CO2 for 24 h followed by treatment of cells with different concentrations of inhibitors for another 24 hrs and cell proliferation was measured by adding 20 µl of MTT (thiazolyl blue tetrazolium bromide) dye (5 mg/ml in sterile phosphate-buffered saline) per well. The plates were then incubated for further 4 hrs at 37 °C in a humidified chamber containing 5% CO2. Formazan crystals formed due to reduction of dye by viable cells in each well were dissolved in 150 mL dimethyl sulfoxide, and absorbance read at 492 nm. The absorption values were expressed as the cell proliferation rate (%), according to the control group as 100%.

## Electronic supplementary material

Supplementary Information 
