# Supplementary material for: Potential inhibitors designed against NDM-1 type metallo-β-lactamases: an attempt to enhance efficacies of antibiotics against multi-drug-resistant bacteria
Source: Sci Rep. 2017 Aug 23;7:9207. doi: 10.1038/s41598-017-09588-1 (PMC5569068; doi:10.1038/s41598-017-09588-1)
Supplement: Supplementary file 1 — Supplementary Information [file 41598_2017_9588_MOESM1_ESM.doc]

**Potential inhibitors designed against NDM-1 type metallo-β-lactamases : an attempt to enhance efficacies of antibiotics against multi-drug-resistant bacteria**

Asad U Khan^1*#^, Abid Ali^1#^, Danishuddin^1^, Gaurava Srivastava^2^, Ashok Sharma^2^

^1^Interdisciplinary Biotechnology Unit, Aligarh Muslim University Aligarh, India 202002, India.

^2^Biotechnology Division, CSIR-CIMAP , Lucknow-226015, India.

Author for correspondence*: Prof. Asad U Khan, Interdisciplinary Biotechnology Unit, Aligarh Muslim University Aligarh, India 202002, India.


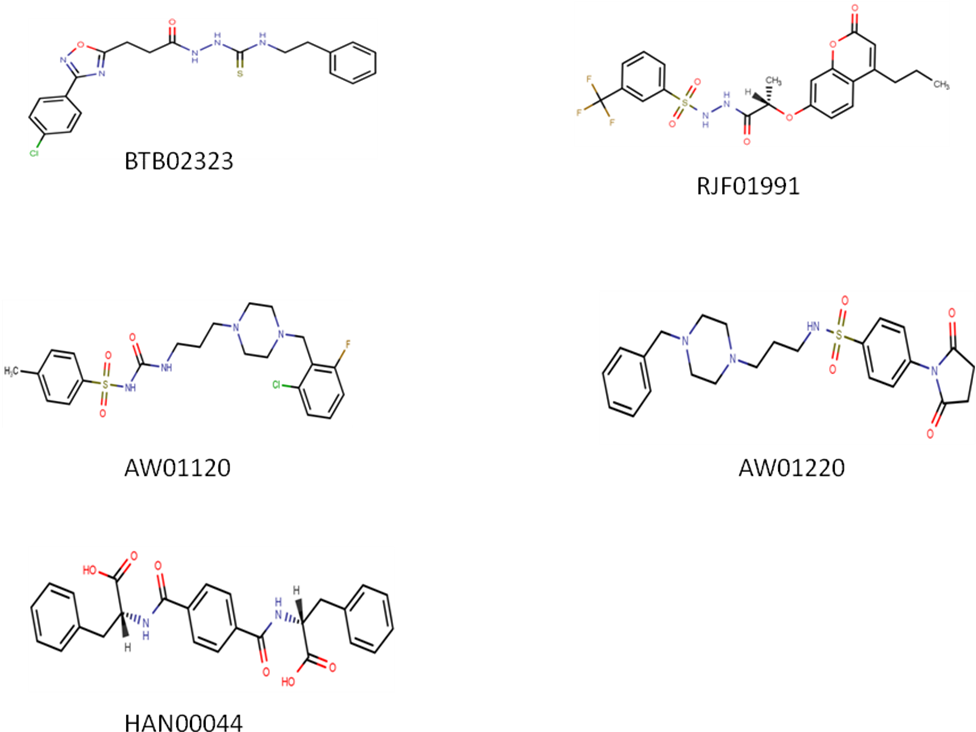


**Figure S 1** 2D structures of selected compounds selected for NDM-1.


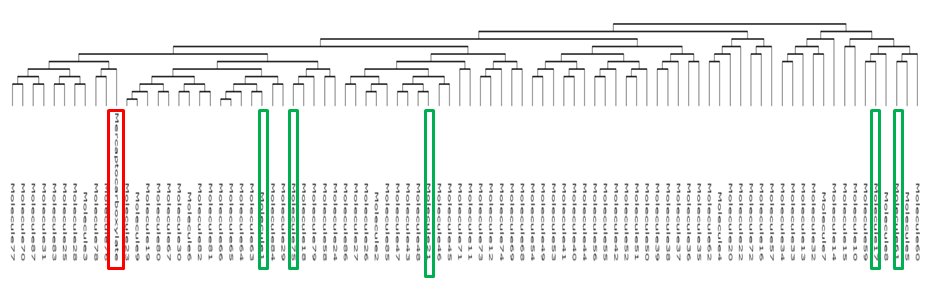


**Figure S2** Hierarchical clustering plot of screened inhibitors against NDM-1.


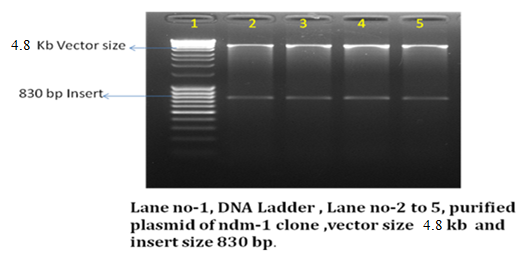


**Figure S 3** Conformation of NDM-1 clone by restriction digestion.


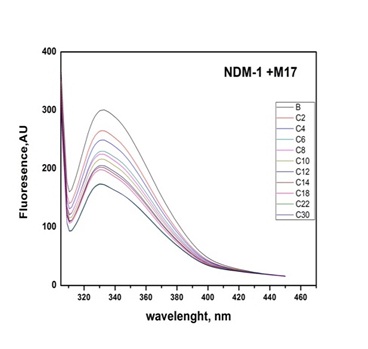


**Figure-S4.** M17 -induced fluorescence quenching of NDM-1. The concentration of NDM-1 was 2 μM, and the concentration of M1 was varied from 0 to 34 μM in a successive increment of 2 μM.


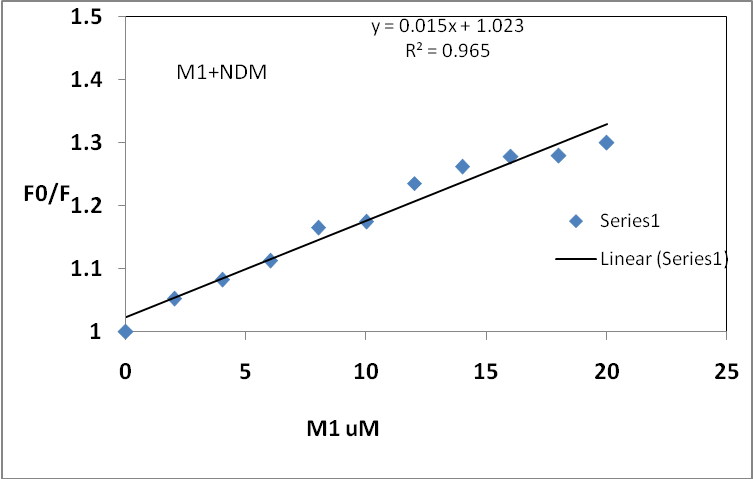


**Figure S5**: .M1-induced fluorescence quenching of NDM-1 at 298 K. shows the Stern−Volmer plot for NDM-1. The concentration of NDM-1 was 2 μM in 20 mM sodium phosphate buffer at pH 7.0.


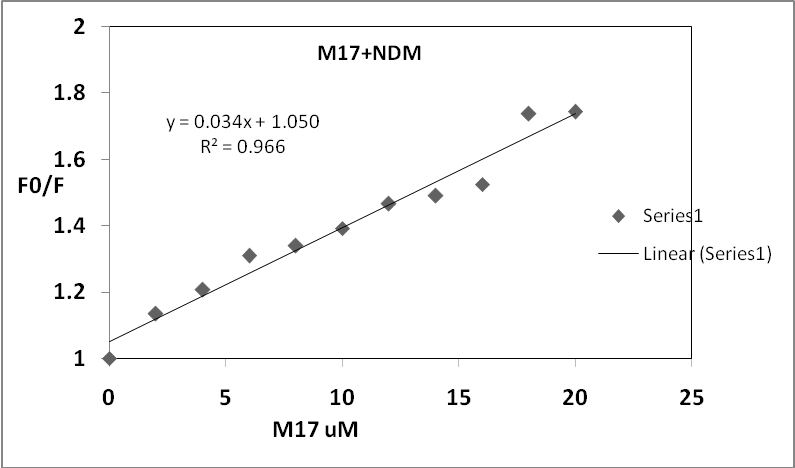


**Figure S6**: .M17-induced fluorescence quenching of NDM-1 at 298 K. shows the Stern−Volmer plot for NDM-1. The concentration of NDM-1 was 2 μM in 20 mM sodium phosphate buffer at pH 7.0.


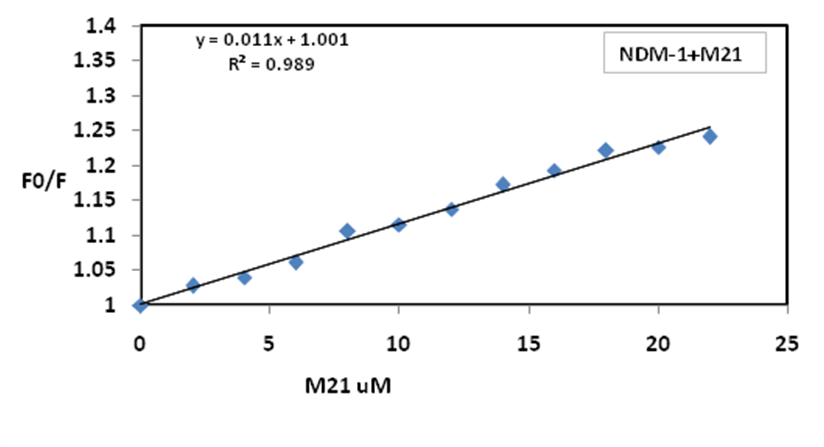


**Figure S7**: .M21-induced fluorescence quenching of NDM-1 at 298 K. shows the Stern−Volmer plot for NDM-1. The concentration of NDM-1 was 2 μM in 20 mM sodium phosphate buffer at pH 7.0.


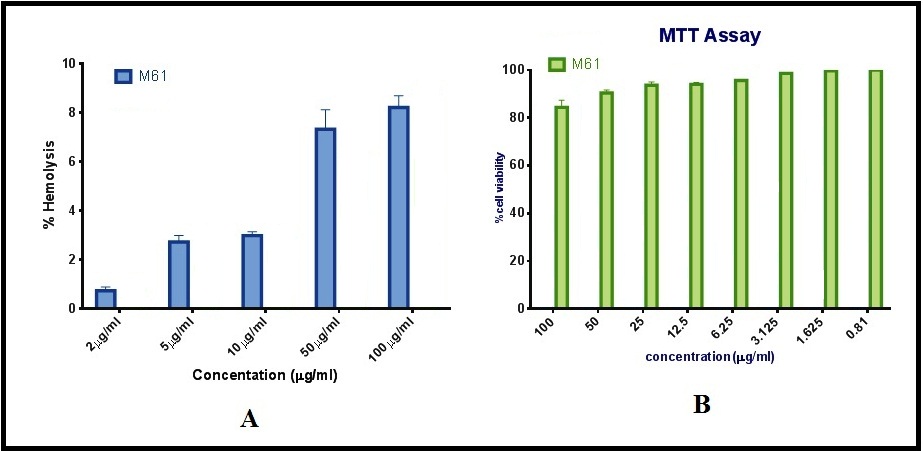


**Figure –S 8** A. Erythrocyte lysis Assay. B.Effect of different concentrations of screened inhibitors M61 on Peripheral blood monocyte cells (PBMCs) for three different time durations (12 hours,24 hours & 48 hours) determined by MTT assay.
